# Significance of High-Mobility Group A Protein 2 Expression in Pancreatic Ductal Adenocarcinoma and Ampullary Adenocarcinoma

**DOI:** 10.5152/tjg.2023.22881

**Published:** 2023-10-01

**Authors:** Damla Oflas, Funda Canaz, İlter Özer, Lütfiye Demir, Ertuğrul Çolak

**Affiliations:** 1 Department of Pathology, Osmangazi University Faculty of Medicine, Eskişehir, Turkey; 2 Department of General Surgery, Osmangazi University Faculty of Medicine, Eskişehir, Turkey; 3 Department of Medical Oncology, Osmangazi University Faculty of Medicine, Eskişehir, Turkey; 4 Department of Biostatistics, Osmangazi University Faculty of Medicine, Eskişehir, Turkey

**Keywords:** HMGA2, epithelial–mesenchymal transition, pancreatic ductal adenocarcinoma, ampullary adenocarcinoma

## Abstract

**Background/Aims:**

Pancreatic and ampullary adenocarcinoma (AAC) are quite resistant to chemotherapy with high metastasis potential. Our study aimed to interpret high-mobility group A protein 2 (HMGA2) expression in benign and precursor pancreatic lesions and pancreatic and ampullary carcinoma and to evaluate its relationship with epithelial–mesenchymal transition (EMT) and clinicopathological parameters.

**Materials and:**

**Methods:** In this study, normal-appearing pancreas, chronic pancreatitis (CP), low- (L) and high (H)-grade pancreatic intraepithelial neoplasia (PanIN), pancreatic ductal adenocarcinoma (PDAC), and AAC were evaluated with the immunohistochemical marker of HMGA2. Vimentin and E-cadherin immunohistochemical stains were applied in PDAC and AAC.

**Results:**

The HMGA2 expression was not detected in normal-appearing pancreas, CP, and L-PanIN. A statistically significant expression was observed in PDAC and H-PanIN (*P* < .001). A statistically significant correlation was found between loss of membranous E-cadherin expression and vimentin positivity and HMGA2 expression (*P* > .05). The HMGA2 expression was observed to increase the risk of disease-related death and decrease overall survival (OS) in AAC and the neoplasia group (*P* = .002 and *P* = .016, respectively). There was no significant difference in OS and risk of death in PDAC (*P* > .05) with respect to HMGA2 positivity.

**Conclusion:**

High-mobility group A protein 2 is a helpful immunohistochemical marker in differentiating CP from PDAC. It also plays a role in EMT and may serve as a potential new prognostic agent and therapeutic target in tumors of the periampullary region, especially AAC.

**Main Points**

- High-mobility group A protein 2 (HMGA2) is expressed in pancreatic carcinoma and high-grade pancreatic intraepithelial neoplasia but not in low-grade pancreatic intraepithelial neoplasia and benign lesions.
- HMGA2 may play a role in pancreatic adenocarcinoma development and has verified the role of epithelial–mesenchymal transition in periampullary carcinomas.
- Immunohistochemical analysis of HMGA2 might be useful in predicting the prognosis of ampullary adenocarcinoma (AAC).
- Future studies may show that HMGA2 may be a potential new therapeutic target in tumors of the periampullary region, especially AAC.

## Introduction

Pancreatic ductal adenocarcinoma (PDAC) is the most common solid neoplasia, accounting for 95% of pancreatic exocrine malignancies.^1^ It is the 14^th^ most common cancer, and the number of cases is increasing worldwide. However, it ranks seventh in cancer deaths and is responsible for 466 003 deaths in 1 year.^2^ The overall survival (OS) rate of patients with PDAC is quite low, with a 5-year OS rate of 9% and a 1-year OS rate of 24%.^3^ On the other hand, it is reported that the incidence of ampullary adenocarcinoma (AAC) is increasing gradually, and the 5-year survival rate is approximately 41%.^4^ The PDAC and AAC are included in periampullary cancers, along with biliary and duodenal cancers. Although clinical findings and treatment overlap, their molecular profile and survival differ significantly.^5^

High-mobility group A proteins (HMGA1 and HMGA2) are different non-histone parts of chromatin that join in many biological events. The HMGA gene family binds to adenosine-thymine (AT)-rich regions in the small groove of DNA with its AT hooks.^6^ They are involved in many biological developments such as DNA damage repair, cell proliferation, stem cell regeneration, epithelial–mesenchymal transition (EMT), metastasis, and tumor invasion.^7^

Epithelial–mesenchymal transition is a biochemical event in which epithelial cancer cells transform into a fibroblastic/mesenchymal character. It has been reported that, as a result of this change, cancer cells gain resistance to apoptosis, increase their migration ability, and acquire more invasive and aggressive features. The characteristic feature of EMT is increased expression of mesenchymal markers such as N-cadherin and vimentin and decreased expression of epithelial markers such as E-cadherin and cytokeratin.^8^

Our study first aimed to establish HMGA2 protein expression in benign and malignant phenotypes of pancreatic exocrine tissue. To clarify the role of HMGA2 in the prognostic assessment of PDAC and AAC, we also evaluated the correlation of HMGA2 protein expression with clinicopathologic parameters and OS and its association with vimentin and E-cadherin.

## Materials and Methods

The study included surgical specimens of 119 patients who underwent subtotal pancreatectomy or pancreaticoduodenectomy for neoplasia or non-neoplasia between 2010 and 2020, available in the archive of the Osmangazi University Department of Pathology. Samples were evaluated according to their histopathologic diagnoses, and normal-appearing pancreas (n = 56), chronic pancreatitis (CP) (n = 86), low-grade pancreatic intraepithelial neoplasia (L-PanIN) (n = 80), high-grade pancreatic intraepithelial neoplasia (H-PanIN) (n = 30), PDAC (n = 57), and AAC (n = 30) study groups were formed. Demographic characteristics, tumor location, and tumor size were recorded. The pathologic preparations were obtained from the archive and re-evaluated in terms of histologic type, differentiation, pathological tumor stage (pT) lymphovascular invasion (LVI), pathological nodal stage (pN), and perineural invasion (PNI).

Data for surviving patients were determined at their final follow-up. Patients with AAC (n = 5) and PDAC (n = 8) who died within the first 1 month due to surgical complications related to the disease and patients with PDAC (n = 2) who died due to other causes were excluded from the OS analyses. 

### Immunohistochemical Staining and Evaluation

Sections of 4 μm were taken from paraffin blocks. Deparaffinization and immunoperoxidase staining of the slides were performed in an automatic staining machine (Dako Omnis, Mannedorf, Switzerland) according to the manufacturer’s rules. The chromogen diaminobenzidine was used. Counterstaining was performed with Harris hematoxylin. After machine processing, it was passed through 96% alcohol and 99% alcohol and xylene. The stained preparations were made ready for evaluation by covering them with a xylene-based slide sealer. HHMGA2 (dilution 1 : 400, rabbit monoclonal, 8179S, Cell Signaling, Massachusetts, USA), E-cadherin (clone NCH-38, rabbit monoclonal, Dako), and vimentin (clone V9, rabbit monoclonal, Dako) (in ready-to-apply form without dilution) were applied to tissues.

Nuclear staining was evaluated with HMGA2 in 200 tumor cells in 5 high-power fields. Staining intensity was evaluated by 2 pathologists in 4 categories: 0 (negative), 1 (weak), 2 (moderate), and 3 (strong). Positive tumor cell staining was scored in 4 grades: 0, 0%; 1, 1%-25%; 2, 26%-49%; 3, 50%-75%; 4, 76%-100%. The overall score was obtained by multiplying the 2 individual scores (0-12). A score of 2 and below was considered negative and a score above 2 was considered positive.^9^

At least 100 tumor cells were evaluated in immunohistochemical analysis with E-cadherin. If 100% of the tumor cells had a similar intensity of membranous staining to the normal-appearing pancreas, it was classified as “intact (no loss).” It was considered as “partial loss” when ≥6% and <99% staining was observed in the tumoral lesion and as “total loss” if <6% staining was observed in the tumoral lesion.^10^

Cytoplasmic staining in any cancer cell was considered positive in the immunohistochemical analysis with vimentin.^11^

### Statistical Analysis

Continuous data are given as mean ± SD. In the comparison of the groups that did not comply with normal distribution, the Mann–Whitney *U* and the Kruskal–Wallis tests were used. To evaluate the statistical association between the clinicopathologic parameters vimentin, E-cadherin, and HGMA2 expression, in 2 × 2 crosstabs, continuity correction, Fisher’s exact test, and Pearson *χ*^2^ tests were performed. Survival curves were obtained using the Kaplan–Meier method, and differences were assessed using the log-rank test. Univariate/multivariate analyses in the Cox regression model were used to show the effect of independent variables on survival. Biostatistical analyses were performed using the Statistical Package for the Social Sciences (version 23.0) (IBM Corp.; Armonk, NY, USA) program. *P* < .05 was considered to indicate statistical significance.

### Ethics Committee Approval

This study was approved by Eskişehir Osmangazi University Faculty of Medicine Non-Invasive Clinical Research Ethics Committee (Date: 16/10 /2020, No: 45). The study protocol conformed to the ethical guidelines of the Declaration of Helsinki. No informed consent was needed because of the retrospective non-interventional study design.

## Results

The mean age of the patients in the PDAC (n = 57) and AAC (n = 30) groups was 65.8 (range: 40-85) and 64.1 years (range:41-81), respectively. The mean greatest dimensions of the tumors were 3.3 ± 1.7 (± SD) cm and 2.5 ± 1.3 (± SD) cm, respectively. The mean age of the entire neoplasia cohort of 87 patients diagnosed with PDAC and AAC was 65.2 (range: 40-85) years. The mean tumor size was 3.0 ± 1.6 (± SD) cm. Clinicopathologic features in the PDAC, AAC, and neoplasia cohort are given in Table 1.

### High-Mobility Group A Protein 2 Expression in Normal Pancreas, Chronic Pancreatitis, Low- and High-Grade Pancreatic Intraepithelial Neoplasia, and Pancreatic Ductal Adenocarcinoma

**Figure 1.**
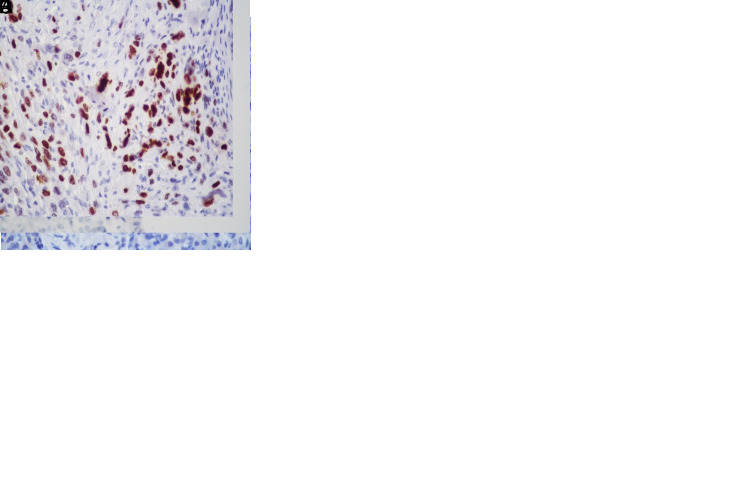
Immunohistochemical staining of high-mobility group A protein 2 (HMGA2) (A-E) in normal-appearing pancreas, chronic pancreatitis, low-grade pancreatic intraepithelial neoplasia (PanIN), high-grade PanIN, and pancreatic cancer groups, respectively. (A, B, C) Negative staining with HMGA2. (D, E) Positive staining with HMGA2. Original magnification, × 400.

Positivity of HMGA2 was demonstrated in 3 (10%) cases in the H-PanIN and 18 (38.1%) cases in PDAC (Figure 1) groups. Positivity of HMGA2 was shown in 3 (10%) cases in the H-PanIN and 18 (38.1%) cases in PDAC (Figure 1) groups. In the normal-appearing pancreas, CP, and L-PanIN groups, HMGA2 was negative (*P* < .001) (Table 2).

### High-Mobility Group A Protein 2 Expression and Clinicopathologic Parameters

**Figure 2.**
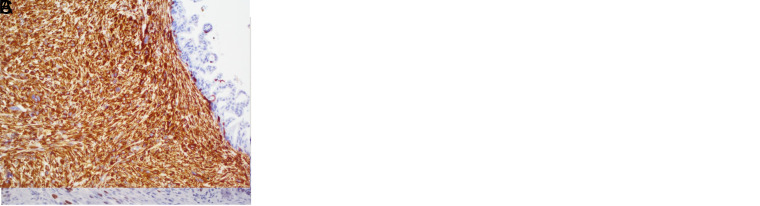
(A) Pancreatic ductal adenocarcinoma case with high-mobility group A protein 2 positive expression. (B) Total loss with E-cadherin, in this case. (C) Vimentin positivity in tumoral cells. Original magnification, × 200.

**Figure 3.**
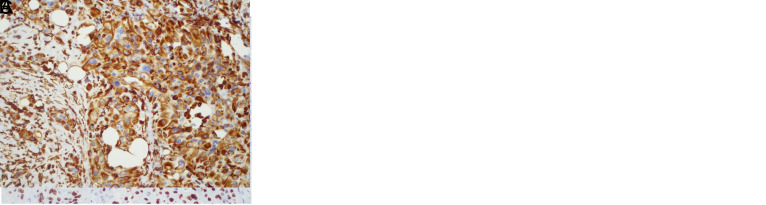
(A) Ampullary adenocarcinoma case with high-mobility group A protein 2-positive expression. (B) Partial loss with E-cadherin in this case. (C) Vimentin positivity in tumoral cells. Original magnification, × 200.

Immunohistochemically, we showed that HMGA2 was expressed only in the nuclei of tumor cells. Of the 57 pancreatic cancers, 18 (31.6%) showed positive HMGA2 expression (Figure 2). HMGA2 was positive in 9 (30%) ampullary cancers (n = 30) (Figure 3). Among the 87 cases in the entire neoplasia cohort, 27 (31%) were positive for HMGA2.

In all groups, HMGA2-positive cases were generally male, with a lower mean age. Tumor size was smaller in positive cases than the negative ones in all groups. HMGA2 positivity was higher in pancreatic localization. HMGA2-positive cases were generally moderately differentiated in all study groups. Lymphovascular invasion and PNI were higher in HMGA2-positive cases. Most HMGA2-positive cases were detected in AAC and PDAC, pT3b, pN1, clinical stage 3a and pT2, pN1, and clinical stage 2b, respectively. However, there was no significant difference between HMGA2-positive and HMGA2-negative cases in terms of gender, age, tumor location, differentiation, size, pT (pathologic tumor) status, pN (pathologic nodal) status, LVI, and PNI parameters (*P* > .05) (Table 3).

### High-Mobility Group A Protein 2 Expression and Overall Survival

**Figure 4.**
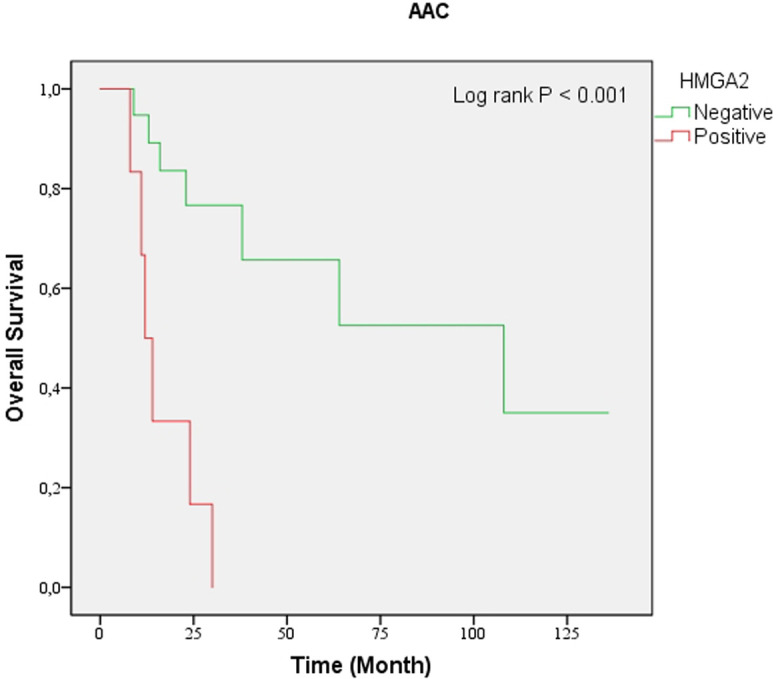
Kaplan–Meier analysis of overall survival according to high-mobility group A protein 2 expression in pancreatic ductal adenocarcinoma (*P* = .597). AAC, ampullary adenocarcinoma; HMGA2, high-mobility group A protein 2.

**Figure 5.**
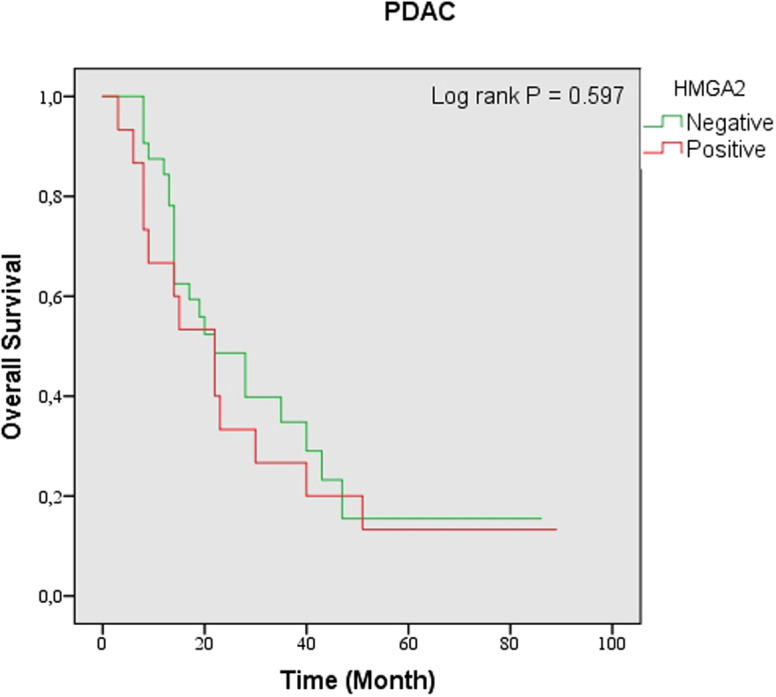
Kaplan–Meier analysis of overall survival according to high-mobility group A protein 2 (HMGA2) expression in ampullary adenocarcinoma. HMGA2 positivity was significantly correlated with shorter overall survival (*P* < .001). HMGA2, high-mobility group A protein 2; PDAC, pancreatic ductal adenocarcinoma.

**Figure 6.**
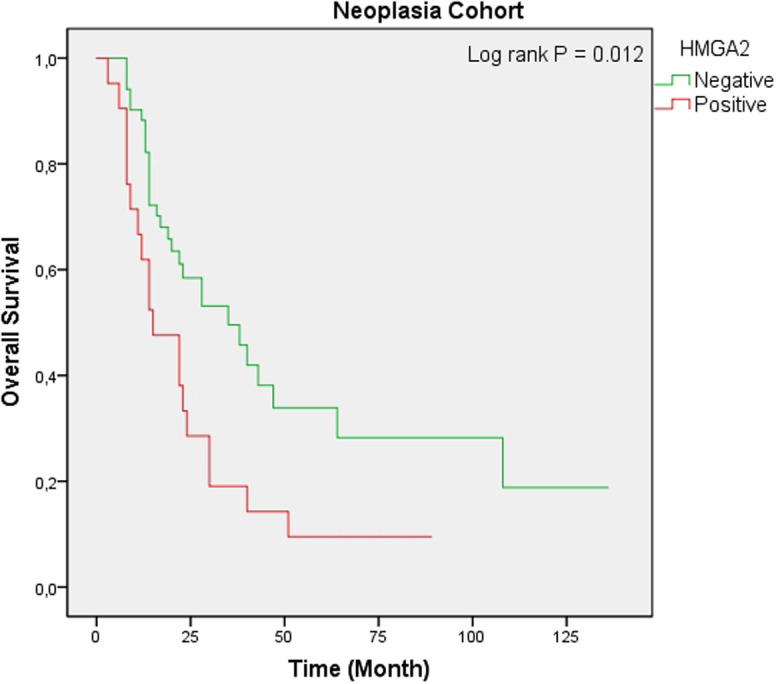
Kaplan–Meier analysis of overall survival according to high-mobility group A protein 2 (HMGA2) expression in the neoplasia cohort. HMGA2 positivity was significantly correlated with shorter overall survival (*P* = .012).

The presence of HMGA2-positive expression was correlated with an importantly shorter OS in the patient groups with AAC (*P* < .001) as well as in the neoplasia group (*P* = .012). However, positive expression of HMGA2 did not associate with OS in the patient group with PDAC (*P* = .597) (Figures 4-6). Univariate analysis in patients with PDAC showed no statistically significant association between OS and sex, age, tumor differentiation, LVI, PNI, and HMGA2 expression (Table 4). Multivariate analysis showed that increasing age [hazard ratio (HR) 1.07, 95% CI (1.01 to 1.13), *P* = .009] and poor tumor differentiation [HR 5.84 95% CI (1.26 to 27.15), *P* = .024) were independently correlated with lower OS.

In univariate analysis, a significant correlation was found between HMGA2 protein expression and OS in patients with AAC (*P* = .002). The most independent predictor of poor OS was PNI; PNI increased the probability of death of patients 10.24 times. The risk ratio of poor OS was 7.93 among patients with positive HMGA2 expression versus those with negative HMGA2 expression. No significant correlation was found between age, sex, tumor differentiation, LVI variables, and OS (*P* > .05). Since convergence could not be achieved in the AAC group, the multivariate analysis method could not be applied. In the univariate analysis, the HMGA2 protein expression was found to increase the risk of disease-related death in the entire neoplasia cohort (*P* = .016). The presence of LVI (*P* = .04) and PNI (*P* = .002) was observed to decrease OS. No significant correlation was found between age, sex, tumor location, differentiation, and OS (*P* > .05) (Table 4). Perineural invasionand LVI were determined to be independent prognostic factors for OS [multivariate analysis: HR 5.71, 95% CI (2.08 to 15.63), *P* = .001 and HR 2.32 95% CI (1.12 to 4.80), *P* = .023, respectively). Age, sex, tumor location, differentiation, and HMGA2 expression were not found to be significantly correlated with OS (*P* > .05).

### Associations of High-Mobility Group A Protein 2, E-cadherin, and Vimentin Expression

E-cadherin was not intact in HMGA2-positive cases in all study groups. In the PDAC study group, we showed 16 partial loss and 2 total loss in HMGA2-positive cases (n = 18). Vimentin was positive in 10 of these 18 cases (55.6%). Partial loss was observed in 8 cases and total loss was observed in 1 case with HMGA2-positive AAC (n = 9). Six of these cases were vimentin positive (66.7%). In the neoplasia cohort, E-cadherin showed a partial loss in 24 and complete loss in 3 in HMGA2-positive cases (n = 27). Vimentin was positive in 16 cases (59.3%) in the neoplasia cohort.

We investigated the relationship between E-cadherin, vimentin, and HMGA expression and found a statistically significant relationship in PDAC, AAC, and all neoplasia groups (Table 5).

## Discussion

Deactivation of the HMGA2 (formerly HMGI/C) gene leads to a dwarf phenotype with characteristic hypoplasia in the mesenchymal tissue; thus, HMGA2 has an important role in mammalian growth and development.^12^ Both HMGA1 and HMGA2 are suppressed or absent in most adult differentiated tissues,^13,14^ although their expression is observed in embryonic tissues.^15,16^ There are studies evaluating HMGA2 protein expression in malignant epithelial tumors such as stomach,^17^ colon,^18^ breast,^19^ lung,^20^ bladder,^21^ skin,^22^ and kidney^23^ carcinomas. These studies report that HMGA2 protein plays a role in tumor development and is not expressed in the normal epithelial tissue. In addition, high expression of HMGA2 has been reported to increase the invasion and metastasis potential of tumor cells.Abe et al^24^ compared HMGA2 protein expression between normal-appearing pancreas, CP, and PDAC groups. Intense and diffuse staining was observed in all cases (17/17) in the PDAC group, and no positivity was found in normal-appearing pancreas (0/6) and CP (0/2). Gundlach et al^25^ evaluated the HMGA2 expression between peritumoral benign ducts (n = 28) and PDAC (n = 106) and reported that expression was significantly higher in PDAC (*P* = .003). Piscuoglio et al^26^ showed that the expression of HMGA1 and HMGA2 was almost absent or low in the normal-appearing pancreas; the expression was increased in PanIN lesions and significantly higher in PDAC, suggesting that these proteins played a role in pancreatic carcinogenesis and the transition to a more malignant phenotype.

In the present study, we examined the expression of HMGA2 protein in the normal-appearing pancreas, low- and high-grade PanIN, and ductal adenocarcinoma. No expression was observed with HMGA2 in the normal-appearing pancreas, CP, and L-PanIN groups, but positive nuclear staining was detected in PDAC and H-PanIN (*P* < .001). Our study is similar to the literature and supports that HMGA2 is a useful molecular marker in the differential diagnosis of pancreatic adenocarcinoma.

In the periampullary tumor group, PDAC and AAC are present together with biliary and duodenal cancers. These tumors have overlapping symptoms and a common treatment (pancreaticoduodenectomy). However, they differ in their survival and biology, including their molecular profile.^5^ Gong et al^9^ evaluated the relationship between positive expression of HMGA2 and gender, age, stage, tumor differentiation, metastatic lymph nodes, and OS in PDAC (n = 60). They observed that the presence of lymph node metastases, poor tumor differentiation, and advanced tumor stage were correlated with HMGA2 protein expression, and increased HMGA2 protein expression was an independent poor prognostic factor. Piscuoligo et al^26^ obtained that HMGA2 expression in PDAC (n : 210) was associated with N status and the degree of differentiation but not with T status and OS. Gundlach et al^25^ evaluated the HMGA2 expression in PDAC as nuclear and cytoplasmic and found a significant correlation between nuclear HMGA2 expression, metastatic lymph nodes, and poor OS.

Although there are various studies evaluating the expression of HMGA2 in PDAC, there are a few studies involving AAC. In our review of previous studies, we identified only 1 study examining the expression of HMGA2 in AAC. Strell et al^27^ investigated the relationship between age, sex, stage, and tumor differentiation with HMGA2 protein expression and its effect on survival in cases of PDAC (n = 253) and AAC (n = 155). In this study, HMGA2 positivity was determined as 56.6% in PDAC and 32.7% in AAC (< .001). They observed that HMGA2 expression was associated with poor tumor differentiation in patients with PDAC cases and increased age in patients with AAC. In addition, HMGA2 positivity in AAC cases was generally found in moderately differentiated tumors, but no statistically significant difference was found. They showed that HMGA2 expression in both PDAC and AAC was an independent prognostic factor for OS in multivariate analysis. In our study, HMGA2 positivity in pancreatic and ampulla tumors was 66.7% and 33.3%, respectively (*P* > .05). The HMGA2-positive cases were generally moderately differentiated in the study groups. Lymphovascular invasion and PNI were higher in HMGA2-positive cases. Most HMGA2-positive cases were detected in AAC and PDAC, pT3b, N1, clinical stage 3a and pT2, N1, and clinical stage 2b, respectively. However, we detected no significant correlation between HMGA2 protein expression and age, sex, tumor size, tumor differentiation, LVI, PNI, stage, and pathologic T and N status in patients with PDAC and AAC (*P* > .05). There was also no statistically significant relationship between HMGA2 expression and OS in PDAC. However, unlike PDAC, the univariate regression analysis revealed that HMGA2 protein expression increased the risk of disease-related death in patients with AAC, and OS decreased in HMGA2-positive cases in Kaplan–Meier survival analysis. Our findings suggest that immunohistochemical analysis of HMGA2 may be useful in predicting prognosis in AAC. However, the disadvantage of our study is that the multivariate analysis method could not be applied because convergence could not be achieved in the AAC group.

We also investigated patients with PDAC and AAC with periampullary region tumors as a neoplasia cohort. We found no significant relationship between HMGA2 expression and clinicopathologic variables (*P* > .05). Univariate analysis showed that LVI, PNI, and HMGA2 expression (*P* = .040, *P* = .002, and *P* = .016, respectively) were correlated with OS. In multivariate regression analysis, the presence of LVI and PNI was found to be an independent prognostic factor (*P* = .023 and *P* = .001, respectively). In the Kaplan–Meier curves, it was determined that the OS was decreased in HMGA2-positive cases (*P* = .012). The limitation of our study is the number of cases, and more precise results can be given in larger series.

Many researchers studied EMT in the 1990s for its association with invasion, growth, and metastasis of cancer cells.^28^ Cadherin-mediated cell adhesion plays an important role in the early embryonic period when numerous phenotypic changes occur at the EMT. The transition to the fibroblastic nature, which lets cells to detach from epithelial tissue and migrate easily, is associated with loss of E-cadherin. This is an important event during gastrulation movements and neural crest formation but is also thought to play a principal role in the early stages of invasion and metastasis of carcinoma cells.^29^

Also, EMT shows upregulation of extracellular matrix components (collagens α1 and α2) and mesenchymal immunohistochemical stains (i.e., alpha-smooth muscle actin, vimentin, N-cadherin, and S100A4). Epithelial–mesenchymal transition plays a role in the most challenging traits of pancreatic cancer cells, which are their invasiveness and drug resistance. Furthermore, EMT-associated molecular pathways are responsible for the metastatic potential of pancreatic cancer cells and their tumor-promoting processes ranging from initiation to desmoplasia and cancer stem cells. Current and future studies on pancreatic cancer and EMT show promising therapeutic targets.^8^ Therefore, we investigated the correlation between HMGA2 expression and E-cadherin and vimentin expression. In our study, we found a statistically significant relationship between loss of E-cadherin expression and vimentin positivity in all groups. Kocsmar et al^30^ studied the prognostic value of tumor budding and E-cadherin expression, one of the EMT markers, in tumors of the periampullary region. They found that membranous E-cadherin expression was decreased in budding tumor cells and had a negative effect on OS in both cases. In their study including 14 patients with pancreatic adenocarcinoma, Watanabe et al^7^ observed that HMGA2 and vimentin expression increased while E-cadherin expression decreased using Western blot analysis when the RAS/MEK pathway was inhibited. They reported that HMGA2, a potential treatment target in pancreatic cancer cells, had a role in RAS-mediated EMT.

HMGA2 is expressed in pancreatic carcinoma and H-PanIN but not in L-PanIN and benign lesions. The HMGA2 expression may assist in the histopathological differentiation of pancreatic carcinoma. Our findings demonstrated that HMGA2 may be involved in the development of pancreatic cancer cells and confirmed the role of EMT in periampullary carcinomas. Immunohistochemical evaluation of HMGA2 may help to determine the prognosis of AAC. Future studies may show that HMGA2 may be a potential new therapeutic target in tumors of the periampullary region (especially AAC).

## Figures and Tables

**Table 1.** Clinicopathological Characteristics of Neoplasia Cohort, Pancreatic Ductal Adenocarcinoma, and Ampullary Adenocarcinoma

Clinicopathological Characteristics	Neoplasia Cohort, n (%)	PDAC, n (%)	AAC, n (%)
Gender			
Female	45 (51.7)	32 (56.1)	13 (43.3)
Male	42 (48.3)	25 (43.9)	17 (56.7)
Differentiation			
Well	8 (9.2)	4 (7.0)	4 (13.3)
Moderate	61 (70.1)	39 (68.4)	22 (73.3)
Poor	18 (20.7)	14 (24.6)	4 (13.3)
PNI			
Absent	67 (77)	54 (94.7)	13 (43.3)
Present	30 (23)	3 (5.3)	17 (56.7)
LVI			
Absent	60 (69)	37 (64.9)	23 (76.7)
Present	27 (31)	20 (35.1)	7 (23.3)
pT stage^*^			
T1		0 (0)	1 (3.3)
T1a		0 (0)	1 (3.3)
T1b		0 (0)	2 (6.7)
T1c		10 (17.5)	
T2		34 (59.6)	7 (23.3)
T3		12 (21.1)	
T3a		0 (0)	2 (6.7)
T3b		0 (0)	17 (56.7)
T4		1 (1.8)	
pN stage^*^			
N0		18 (31.6)	10 (33.3)
N1		26 (45.6)	14 (46.7)
N2		13 (22.8)	6 (20.0)
Clinical stage^*^			
I			
IA		8 (14.0)	1 (3.3)
IB		9 (15.8)	6 (20.0)
II			
IIA		3 (5.3)	1 (3.3)
IIB		25 (43.9)	1 (3.3)
III		12 (21.1)	0 (0)
IIIA		0 (0)	15 (50.0)
IIIB		0 (0)	6 (20.0)
IV		0 (0)	0 (0)
Survival status^**^			
Alive	24 (27.5)	12 (21)	12 (40)
Exitus (due to illness)	48 (55.1)	35 (61.4)	13 (43.3)
Exitus (due to other reasons)	15 (17.2)	10 (17.5)	5 (16.6)

AAC, ampullary adenocarcinoma; LVI, lymphovascular invasion; PDAC, pancreatic ductal adenocarcinoma; PNI, perineural invasion.

^*^Not specified in the neoplasia cohort as assessment differs.

^**^Calculated on 47 PDAC and 25 AAC cases with clinical follow-up data available.

**Table 2.** Comparison of High-Mobility Group A Protein 2 Expression Between Non-neoplastic and Neoplastic Groups

HMGA2 Expression	Normal	CP	PanIN (Low)	PanIN (High)	PDAC	*P*
	n (%)	n (%)	n (%)	n (%)	n (%)	
Negative	57 (100)	86 (100)	80 (100)	27 (90)	39 (68.4)	**< .001**
Positive	0 (0)	0 (0)	0 (0)	3 (10)	18 (31.6)	

Bold items highlight *P* < .05.

CP, chronic pancreatitis; HMGA2, high-mobility group A protein 2; PanIN, pancreatic intraepithelial neoplasia; PDAC, pancreatic ductal adenocarcinoma.

**Table 3.** Associations Between High-Mobility Group A Protein 2 Expression and Clinicopathological Characteristics in the Neoplasia Cohort, Pancreatic Ductal Adenocarcinoma, and Ampullary Adenocarcinoma

HMGA2 Expression	Neoplasia cohort (n = 87)			PDAC (n = 57)			AAC (n = 30)	
	Negative	Positive	*P*	Negative	Positive	*P*	Negative	Positive
*P*
	n (%)	60 (68.9)	27 (31)		39 (68.4)	18 (31.6)		21 (70)	9 (30)	
Age, mean ± SD	65.63 ± 10.2	64.3 ± 9.9	.572	66.31 ± 9.7	64.7 ± 8.6	.558	64.3 ± 11.2	63.4 ± 12.7	.842
Gender			.108			.135			.691
Female	35 (58.3)	10 (37)		25 (64.1)	7 (38.9)		10 (47.6)	3 (33.3)	
Male	25 (41.7)	17 (63)		14 (35.9)	11 (61.1)		11 (52.4)	6 (66.7)	
Localization			1.000						
Pancreas	39 (65)	18 (66.7)							
Ampulla	21 (35)	9 (33.3)							
Diameter (cm), mean ± SD	3.1 ± 1.69	2.9 ± 1.48	.865	3.4 ± 1.74	3.0 ± 1.60	.228	2.4 ± 1.41	2.7 ± 1.25	.372
Differentiation			.113			.224			.549
Well	5 (8.3)	3 (11.1)		3 (7.7)	1 (5.6)		2 (9.5)	2 (22.2)	
Moderate	46 (76.7)	15 (55.6)		29 (74.4)	10 (55.6)		17 (81)	5 (55.6)	
Poor	9 (15)	9 (33.3)		7 (17.9)	7 (38.9)		2 (9.5)	2 (22.2)	
LVI			.66			1.000			.393
Absent	20 (33.3)	7 (25.9)		14 (35.9)	6 (33.3)		6 (28.6)	1 (11.1)	
Present	40 (66.7)	20 (71.4)		25 (64.1)	12 (66.7)		15 (71.4)	8 (88.9)	
PNI			.136			.544			.123
Absent	17 (28.3)	3 (11.1)		3 (7.7)	0 (0)		14 (66.7)	3 (33.3)	
Present	43 (71.7)	24 (88.9)		36 (92.3)	18 (100)		7 (33.3)	6 (66.7)	
pT stage^*^						.374			.814
pT1				0 (0)	0 (0)		1 (4.8)	0 (0)	
pT1a				0 (0)	0 (0)		1 (4.8)	0 (0)	
pT1b				0 (0)	0 (0)		1 (4.8)	1 (11.1)	
pT1c				5 (12.8)	5 (27.8)		0 (0)	0 (0)	
pT2				23 (59)	11 (61.1)		6 (28.6)	1 (11.1)	
pT3				10 (25.6)	2 (11.1)		0 (0)	0 (0)	
pT3a				0 (0)	0 (0)		1 (4.8)	1 (11.1)	
pT3b				0 (0)	0 (0)		11 (52.4)	6 (66.7)	
pT4				1 (2.6)	0 (0)		0 (0)	0 (0)	
pN stage^*^						.889			.868
pN0				13 (33.3)	5 (27.8)		8 (38.1)	2 (22.2)	
pN1				17 (43.6)	9 (50)		9 (42.9)	5 (55.6)	
pN2				9 (23.1)	4 (22.2)		4 (19)	2 (22.2)	
Clinical stage^*^						.517			.719
Ia				4 (10.3)	4 (22.2)		1 (4.8)	0 (0)	
Ib				8 (20.5)	1 (5.6)		5 (23.8)	1 (11.1)	
IIa				2 (5.1)	1 (5.6)		0 (0)	1 (11.1)	
IIb				16 (41)	9 (50)		1 (4.8)	0 (0)	
III				9 (23.1)	3 (16.7)		0 (0)	0 (0)	
IIIa				0 (0)	0 (0)		10 (47.6)	5 (55.6)	
IIIb				0 (0)	0 (0)		4 (19)	2 (22.2)	

AAC, ampullary adenocarcinoma; HMGA2, high-mobility group A protein 2; LVI, lymphovascular invasion; PDAC, pancreatic ductal adenocarcinoma; PNI, perineural invasion.

^*^Not specified in the neoplasia cohort as assessment differs.

**Table 4.** Univariate Analyses of Prognostic Variables Associated with the Overall Survival of All Cancer Patients

Variables	Univariate Analysis					
	Neoplasia Cohort (n = 72n = 72)		PDAC (n = 47)		AAC (n = 25)	
	HR (95% Cl)	*P*	HR (95% Cl)	*P*	HR (95% Cl)	*P*
Age	0.99 (0.96 to 1.02)	.924	1.02 (0.98 to 1.06)	.320	0.95 (0.90 to 1.01)	.112
Gender						
Male	1		1		1	
Female	1.275 (0.71 to 2.26)	.406	0.54 (0.27 to 1.06)	.076	1.13 (0.37 to 3.48)	.824
Tumor localization						
Pancreas	1					
Ampulla	0.52 (0.27 to 1.02)	.058				
Tumor differentiation						
Well	*	.038	*	.221	*	.132
Modarate	1.72 (0.61 to 4.85)	.303	2.22 (0.60 to 8.18)	.227	0.89 (0.14 to 5.51)	.909
Poor	0.72 (0.27 to 1.88)	.507	1.18 (0.35 to 4.00)	.784	0.27 (0.05 to 1.40)	.120
Lymphovascular invasion						
No	1		1		1	
Yes	2.02 (1.03 to 3.95)	**.040**	1.96 (0.92 to 4.20	.080	3.13 (0.66 to 14.88)	.150
Perineural invasion						
No			1		1	
Yes	4.26 (1.67 to 10.89)	**.002**	1.37 (0.32 to 5.74)	.664	10.24 (2.13 to 49.28)	**.004**
HMGA2						
Negative			1			
Positive	2.04 (1.14 to 3.66)	**.016**	1.20 (0.59 to 2.40)	.609	7.93 (2.20 to 28.62)	**.002**

Bold items highlight *P* < 0.05.

AAC, ampullary adenocarcinoma; HMGA2, high-mobility group A protein 2; HR, hazard ratio; PDAC, pancreatic ductal adenocarcinoma.

**Table 5.** Association of E-cadherin, Vimentin, and High-Mobility Group A Protein 2 Protein Expression in Neoplasia Cohort, Pancreatic Ductal Adenocarcinoma, and Ampullary Adenocarcinoma

	Neoplasia Cohort (n = 87) HMGA2 Expression			PDAC (n = 57n = 57) HMGA2 Expression			AAC (n = 30n = 30) HMGA2 Expression		
	Negative	Positive	*P*	Negative	Positive	*P*	Negative	Positive	*P*
n (%)	60 (69)	27 (31)		39 (68.4)	18 (31.6)		21 (70)	9 (30)	
E-cadherin			**< .001**			**.006**			**< .001**
No loss	29 (48.3)	0 (0)		14 (35.9)	0 (0)		15 (71.4)	0 (0)	
Partial loss	30 (50)	24 (88.9)		24 (61.5)	16 (88.9)		6 (28.6)	8 (88.9)	
Total loss	1 (1.7)	3 (11.1)		1 (2.6)	2 (11.1)		0 (0)	1 (11.1)	
Vimentin			**< .001**			**.005**			**.042**
Negative	49 (81.7)	11 (40.7)		33 (84.6)	8 (44.4)		16 (76.2)	3 (33.3)	
Positive	11 (18.3)	16 (59.3)		6 (15.4)	10 (55.6)		5 (23.8)	6 (66.7)	

Bold items highlight *P* < 0.05.

AAC, ampullary adenocarcinoma; HMGA2, high-mobility group A protein 2; PDAC, pancreatic ductal adenocarcinoma.
